# Nationwide experiences with youth-targeted smoking and nicotine product cessation

**DOI:** 10.18332/tpc/169498

**Published:** 2023-08-04

**Authors:** Sofie Kirstine Bergman Rasmussen, Charlotta Pisinger

**Affiliations:** 1 Center for Clinical Research and Prevention, Frederiksberg, Denmark; 2 Department of Public Health, University of Copenhagen, Denmark

**Keywords:** smoking cessation, adolescents, young adults, youth, Denmark, nicotine products

## Abstract

**INTRODUCTION:**

Most adolescent and young adult (youth) smokers and users of novel nicotine products wish to quit. Little is known, at a population level, about youth cessation activities, and the counselor’s experiences in working with youth smoking and nicotine product cessation.

**METHODS:**

A questionnaire was mailed to all 98 municipalities in Denmark on 31 October 2022. Youths were defined as those aged 16–25 years. The participation rate was 96% (n=94). Simple descriptive statistics were performed.

**RESULTS:**

This survey explored youth-targeted smoking and nicotine product cessation activities and ex-periences from municipality counselors across the whole nation. Overall, 60% of the Danish municipal counselors had low/very low/no personal experience with youth cessation interventions, 89% found it dif-ficult to work with youth counseling, 90% found it difficult to recruit youth to nicotine cessation services, and only 25% of the active municipalities were described as highly experienced. A higher percentage of the highly experienced municipalities reported that they share the responsibility of recruitment to cessation services with schools, counsel youths in separate groups from adults, and have good experiences with online counseling.

**CONCLUSIONS:**

This Danish nationwide survey showed that even in a country with very well-organized and free-of-charge cessation counseling programs, very few municipalities give assistance to youth, and most find it difficult to work with youth. Cessation services have been designed for adult smokers and seem to have failed to meet the needs of young smokers and users of novel nicotine products, at least in Denmark. There is an urgent need for research on how to effectively recruit youth to cessation services, and what works to help youth quit.

## INTRODUCTION

Smoking has a negative impact on both physical and mental health^1^ in adolescence and early adulthood, and young smokers of at least five cigarettes per day have a more than fifty percent excess risk of missing more than three school days per month due to illness^2^. Many young people perceive daily life with nicotine abstinence as stressful. The cognitive and affective processes are disrupted^3^, social attitudes stigmatize smokers^4^, smoking bans make it difficult to smoke, and cigarettes are very costly for adolescents, which often results in financial stress^5^.

More and more countries across the world are progressing to high levels of implementation of the World Health Organization’s (WHO) MPOWER tobacco control package, which has led to decreasing smoking rates in many countries, including among young people^6^. However, in the last decade, a large number of novel nicotine products have emerged on the market, and nicotine products such as e-cigarettes, smokeless tobacco, nicotine pouches and heated tobacco are now very popular among adolescents and young adults^7^. Further, there has been an increase in the proportion of young people who use more than one nicotine-containing product, i.e. dual or triple use^8^. There is increasing evidence that nicotine is harmful to the developing brain as it can disrupt the formation of brain circuits that control attention, learning and susceptibility to addiction^9^.

In Denmark, 35% of boys/young men and 27% of girls/young women in the general upper secondary schools use at least one nicotine product daily^10^. Recently, there has been a steep increase in the use of smokeless nicotine products (mostly nicotine pouches) in Danish youth; more than 11% of people aged 15–29 years use a smokeless nicotine product daily or occasionally^11^.

In the UK among tobacco smokers and users of nicotine products, 80% of people aged 12–19 years had already made one attempt to quit^12^. In Denmark, the same percentage, 80%, of those aged 16–24 years report that they want to quit smoking^13^. Eighty-six percent of Danish female smokers aged 16–24 years want to quit, which is the highest proportion in the whole population^13^. Further, more than 70% of daily users aged 16–29 years of smokeless nicotine products have considered stopping in the last month^14^.

However, even though adolescent smokers are very interested in quitting, only a fraction, approximately 3–7%, report being abstinent one year after an unassisted attempt to quit^15^. Smoking cessation services are one of the effective MPOWER measures. Denmark has developed high-quality smoking cessation services targeted at all smokers and the country recently received a high score (8/10) on the European Tobacco Control Scale, when it came to the treatment of smokers^16^. The nationwide, free-of-charge gold standard program consists of smoking cessation behavioral counseling courses over five to six sessions, most often in groups, and the program has proved to be very effective^17^. Unfortunately, adolescents and young adults make little use of these municipal evidence-based smoking cessation services^18^.

In the last few decades, there has been a research interest when it comes to the development and testing of smoking cessation interventions targeted at adolescents and young adults (youth)^19^. However, when the project money runs out there typically are no resources/no manpower to implement the interventions on a larger scale, and little is known about what the real-world experiences are. Further, there is great demand for knowledge from Danish cessation counselors and international public health researchers about cessation from nicotine products (such as nicotine pouches and e-cigarettes), and real-world experiences with the treatment of youth highly dependent on nicotine with dual or triple use of different nicotine products.

Our study aimed to describe practices and experiences with smokers aged 16–25 years (youth) and users of novel nicotine products from the perspective of municipal counselors, at a national level in a small Scandinavian country (population of 5.857 million) with free access to tobacco cessation counseling. Further, we wanted to investigate if there were differences in answers according to counselors’ level of self-reported experience of smoking/nicotine cessation in youth. To our knowledge, there are no previous studies describing nationwide real-world practices and experiences with youth-targeted smoking and nicotine cessation activities.

## METHODS

We performed a cross-sectional study through a national survey targeting municipality cessation counselors. The questionnaire was developed for this study, tested by three smoking cessation counselors, and set up in SurveyXact. Municipal smoking cessation counselors across the whole of Denmark were identified through the municipalities’ websites and later confirmed via phone calls. The questionnaire was emailed to smoking cessation counselors in all 98 municipalities in Denmark on 31 October 2022. As the larger municipalities have several smoking cessation counselors, we encouraged the counselors to complete the questionnaire together, if relevant. We sent two reminders by email, within three weeks. After three weeks SBR tried to contact all non-responders by telephone, up to three times. Most municipalities were reached after the first two calls. Six questionnaires were completed by SBR, by telephone, and guided by the counselor.

The questionnaire covered the following topics: the municipality’s level of involvement in youth-targeted smoking-and nicotine cessation activities, counselors’ qualifications, recruitment of youth, organization of the youth-targeted cessation counseling, the youths’ motivation to quit, drop-out levels during the counseling program, communication methods, perceived dependency of the youths, use of nicotine replacement therapy and perception of success with smoking and novel nicotine product cessation counseling. ‘Youths’ were at the beginning of the questionnaire defined as ‘persons aged 16–25 years’.

Most of the questions had four response categories. In this article, we dichotomized most answers (e.g. very good/good vs bad/very bad or very high/high vs low/very low). The size of the municipality (large ≥70000 citizens, small <70000) was included in the dataset.

### Statistical analysis

Simple descriptive statistics were performed. To investigate possible differences in responses according to the level of self-reported experiences in youth cessation counseling, we classified municipalities into two categories: ‘highly experienced’ and ‘less experienced’. A municipality was identified as highly experienced if the municipality reported high/very high levels of experience with smoking cessation and/or nicotine product cessation for youths, and if the counselors answering the questionnaire reported high/very high levels of personal experience with cessation counseling for youths.

Descriptive analyses were performed in SAS Version 8.3 (64-bit).

## RESULTS

The participation rate was high, 96% (n=94); 71 (76%) out of the 94 Danish municipalities completed the questionnaire fully and 23 (24%) partly, depending on their level of experience with cessation in youth (Table 1). Eight municipalities with no youth experience at all (neither in smoking cessation nor nicotine product cessation) were not asked to complete the rest of the questionnaire, and another eight respondents reported no personal counseling experience and were hence not able to answer most of the questionnaire and were therefore excluded from the analysis. Thus, the results presented in Tables 2, 3, and 4 are based on the 78 municipalities reporting very high, high, low, or very low levels of experience (=active) in smoking and/or nicotine product cessation in youths.

**Table 1:** Municipalities’ overall level of experience with youth-targeted cessation of smoking and nicotine products. A national survey performed in Denmark in 2022

	*Number of municipalities (N=94) n*	*%*
**The municipality’s level of experience with smoking cessation counseling in youth**		
Very high/high	18	19.1
Low/very low	67	71.3
Noa	9	9.6
**The municipality’s level of experience with nicotine product cessation counseling in youth**		
Very high/high	18	20.2
Low/very low	58	61.7
Noa	18	19.1
**Counselor’s personal level of experience with youth cessation interventions**		
Very high/high	34	39.5
Low/very low	44	51.2
Nob	8	9.3
**Counselors’ perception of success with municipality’s smoking cessation interventionsc**		
Very good/good	30	42.3
Not good/bad	41	57.7
**Counselors’ perception of success with nicotine product cessation interventionsc**		
Very good/good	32	51.6
Bad/very bad	30	48.4

a Municipalities with no experience with youth smoking cessation and nicotine product counseling were not asked to answer the rest of the questionnaire.

b Municipalities with counselors with no personal experience were excluded from further analyses.

c Answers to the question: ‘How well do you perceive you succeed in helping young people stop smoking/using nicotine products?’.

**Table 2:** Experiences with youth-targeted cessation of smoking and nicotine products, and recruitment of youth among municipalities that actively work with youth cessation

	*Highly experienced (N=20) n (%)*	*Less experienced (N=58) n (%)*	*Total (N=78) n (%)*
**Municipality size**			
Small (<70000)	9 (45.0)	50 (86.2)	59 (75.6)
Large (≥70000)	11 (55.0)	8 (13.8)	19 (24.4)
**Number of youths in cessation counseling per year, median (IQR), range**			
Small municipalities	20 (12–25), (1–40)	4 (2–10), (0–30)	5 (2–12), (0–40)
Large municipalities	50 (25–60), (10–200)	13 (5.5–15), (0–20)	25 (12–50), (0–200)
**Counselors’ perception of working with youth and smoking and/or nicotine product cessation**			
Very easy/easy	6 (30)	3 (5.3)	9 (11.7)
Difficult/very difficult	14 (70)	54 (94.7)	68 (88.3)
**Counselors’ perception of recruitment of youth to smoking and/or nicotine product cessation**			
Very easy/easy	4 (20)	2 (4.7)	6 (10.5)
Difficult/very difficult	16 (80)	41 (95.3)	57 (90.5)
**Recruitment cooperation with schools** (yes to cooperation: n=57; 61%)			
Schools bear the full responsibility	1 (5)	10 (27.0)	11 (19.3)
Shared responsibility	17 (85)	24 (64.9)	41 (71.9)
Municipality bears the full responsibility	2 (10)	3 (8.1)	5 (8.8)
**Counselors’ perception of youths’ rates of quitting compared with adults’**			
Higher quit rates among youth	4 (21.1)	4 (7.7)	8 (11.3)
Same quit rates	8 (42.1)	17 (32.7)	24 (33.8)
Higher quit rates among adults	7 (36.8)	31 (59.6)	39 (54.9)
Municipality offering youth online counseling			
Yes, only during COVID-19 lockdown	6 (31.6)	12 (23.5)	18 (25.7)
Yes, also after COVID-19 lockdown	4 (21.1)	8 (15.7)	12 (17.1)
No	9 (47.4)	31 (60.8)	40 (57.1)
**Counselors’ experience with online counseling**			
Very good/good	7 (70)	7 (41.2)	10 (37.0)
Mixed/bad/very bad	3 (30)	10 (58.8)	17 (62.9)
Counselors’ perception of youths’ most dominant type of dependence*			
No single dominant type of dependence	8 (42.1)	19 (36.5)	27 (38.0)
Social dependence	6 (31.6)	13 (25.0)	19 (26.8)
Psychological dependence	3 (15.8)	10 (19.2)	13 (18.3)
Physical dependence	2 (10.5)	8 (15.4)	10 (14.1)
Habit	0 (0)	2 (3.8)	2 (2.8)

* Mutually exclusive categories. IQR: interquartile range.

**Table 3:** Experiences with group-based youth-targeted cessation of smoking and nicotine products among municipalities that actively work with youth cessation

	*Highly experienced (N=20) n (%)*	*Less experienced (N=58) n (%)*	*Total (N=78) n (%)*
**Nicotine product cessation included in smoking cessation group-based counseling**			
No: smoking and nicotine product cessation handled separately	4 (21.1)	9 (21.4)	13 (21.3)
Yes: included in the same group-based counseling	15 (78.9)	33 (78.6)	48 (78.7)
**Type of group-based counseling**			
Always/mostly for youth separately	16 (88.9)	29 (63.0)	45 (70.3)
Always/mostly youth mixed with other age groups	2 (11.1)	17 (37.0)	19 (29.7)
**Smoking/nicotine cessation counseling takes part during school hours**			
Yes always/sometimes	18 (90.0)	42 (80.8)	60 (83.3)
No	2 (10.0)	10 (19.2)	12 (16.7)
**Quit date**			
They stop when they are ready (no target quit date)	12 (66.7)	24 (54.5)	36 (58.1)
After the second session	4 (22.2)	11 (25)	15 (24.2)
After the third session or later	2 (11.1)	9 (20.5)	11 (17.7)
**Counselors’ use of educational materials**			
Only use the official educational materials*	2 (12.5)	10 (22.7)	12 (20.0)
Supplement with other materials	10 (62.5)	27 (61.4)	37 (61.7)
Do not use the official educational materials	4 (25.0)	7 (15.9)	11 (18.3)
**Counselors’ perception of youths’ motivation to quit when they start counseling**			
More motivated than adults	2 (10.5)	2 (3.8)	4 (5.6)
No difference between youth and adults	8 (42.1)	19 (36.5)	27 (38.0)
Less motivated than adults	9 (47.4)	31 (59.6)	40 (56.3)
**Drop-out rate**			
Youths drop out more frequently	13 (68.4)	26 (50.0)	39 (54.9)
Same rate for youths and adults	6 (31.6)	24 (46.2)	30 (42.3)
Adults drop out more frequently	0 (0)	2 (3.8)	2 (2.8)
**Counselors’ perception of the effect of interventions to prevent drop-out**			
Major effect	2 (10.5)	2 (1.9)	4 (4.2)
Minor effect	1 (5.3)	11 (21.2)	12 (16.9)
No effect/increase in drop-out/don’t know	16 (84.2)	40 (76.9)	56 (78.9)

* Provided by the Danish Cancer Society.

**Table 4:** Experiences with nicotine replacement therapy (NRT) for youth-targeted cessation of smoking and nicotine products among municipalities that actively work with youth cessation

	*Highly experienced municipalities (N=20) n (%)*	*Less experienced municipalities (N=58) n (%)*	*Total (N=78) n (%)*
**Counselors recommend youth use NRT**			
Yes	14 (73.7)	40 (76.9)	54 (76.1)
No	5 (26.3)	12 (23.1)	17 (23.9)
**Percentage of youth who use NRT,** median (IQR), range	50 (10–70), (1–100)	17.5 (7.5–35), (0–100)	20 (10–50), (0–100)
**Counselors’ age criteria for NRT recommendation**			
Yes Only recommend NRT to youths over age, median ( IQR), range	9 (64.3) 18 (16–18), (15–18)	19 (47.5) 16 (15–18), (15–18)	28 (51.9) 16 (15–18), (15–18)
No	5 (35.7)	21 (52.5)	26 (48.1)
**The municipality provides vouchers for NRT**			
Yes	14 (73.7)	31 (60.8)	45 (64.3)
No	5 (26.3)	20 (39.2)	25 (35.7)
**Counselors’ perception of the necessary doses of NRT in youths compared with adults**			
A little/much higher doses in youth	4 (26.7)	11 (31.4)	15 (30.0)
Same doses	11 (73.7)	18 (51.4)	29 (58.0)
A little lower/much lower doses in youth	0 (0)	6 (17.1)	6 (12.0)
Counselors’ perception of symptoms of overdose of NRT in youths*			
Serious symptoms	1 (6.7)	0 (0)	1 (2.0)
Mild symptoms	3 (20.0)	8 (22.9)	11 (22.0)
Never experienced overdose symptoms	11 (73.3)	27 (77.1)	38 (76.0)
**Counselors’ perception of the effect of NRT on youths who try to quit**			
Very good/good	13 (68.4)	27 (51.9)	40 (56.3)
Bad/very bad	2 (10.5)	8 (15.4)	10 (14.1)
No experience with NRT	4 (21.1)	17 (32.7)	21 (29.6)

* Palpitations, sweating, stomach cramps, nausea, vomiting, drooling, rapid breathing, etc. IQR: interquartile range.

### The municipalities’ overall level of experience with youth-targeted cessation of smoking and nicotine products

As shown in Table 1, 19.1% of the active municipalities had a high or very high level of experience with smoking cessation counseling in youth, whereas 20.2% municipalities had a high or very high level of experience with nicotine product cessation counseling in youth. Nine municipalities (9.6%) had no experience with smoking cessation and 18 (19.1%) had no experience with nicotine product cessation. In eight municipalities (9.3%), counselors with no personal experience in youth cessation counseling answered the questionnaire, and 51.6% of the municipality counselors reported low/very low personal experience. In all, 42.3% of the active municipalities reported having perceived good or very good smoking cessation results and 51.6% reported that they had perceived good or very good results with cessation of nicotine products in youth.

### Danish municipalities’ experience with youth-targeted cessation of smoking and nicotine products, and recruitment of youth (Table 2)

As shown in Table 2, 20 (25.6%) of the active municipalities were categorized as highly experienced (14 had very high/high experiences with both smoking and nicotine product cessation, 3 had very high/high experience with smoking cessation only, and 3 had very high/high experience with nicotine product cessation only).In all, 86.2% of the less experienced municipalities were small municipalities, compared to the highly experienced municipalities, of which 55% were large. Among the active municipalities, the highly experienced municipalities (both small and large) reported a higher number of youths in smoking cessation activities per year.

Among the municipalities that cooperate with schools regarding recruitment, 85% of the highly experienced municipalities reported sharing the responsibility of recruitment with the schools, compared to 64.9% among the less experienced municipalities.

Most of the experienced municipalities (42.1%) reported that it was their perception that abstinence rates were the same among youths and adults, compared to the less experienced municipalities where most (59.6%) reported that it was their perception that abstinence rates were lower in youths than in adults. Compared to the less experienced municipalities, more of the highly experienced municipalities had experience with online counseling of youth both during and after the COVID-19 lockdown and reported better experiences with online counseling.

When asked about the perception of the dominant type of dependence among youth, the most common answer for both the highly experienced and less experienced municipalities was that there was not one dominant type of dependence; the second most common answer for all municipalities was that it was social dependence.

### Smoking cessation counselors’ perception of the most motivating factors for youths’ wish to quit

**Figure 1:**
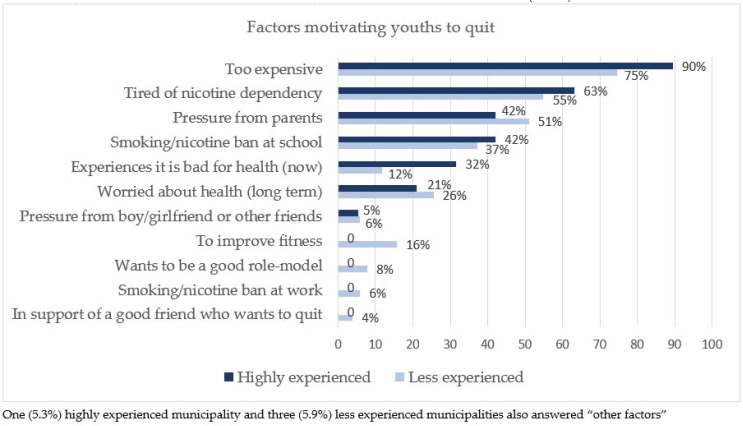
Danish municipalities’ perception of the most important factors motivating youths to quit in percentages

When asked about their perception of the three most important motivating factors for youth to quit, the majority of both the highly experienced (90%) and less experienced (75%) municipalities reported that it was because it was too expensive (Figure 1). Health-related issues were reported as the perceived fifth to seventh most important motivating factors; 32% of highly experienced municipalities perceived that the young people experiencing that smoking/using nicotine products is bad for their health at the present time was a factor motivating youths to quit, compared to only 12% of the less experienced municipalities.

### Smoking cessation counselors’ perception of the most important themes discussed when youth try to quit

**Figure 2:**
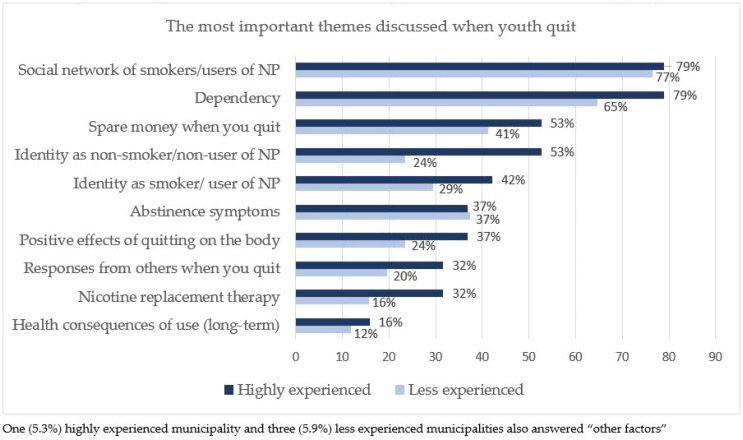
Danish municipalities’ perception of the most important themes discussed when youth try to quit, in percentages

When the counselors were asked about their perception of the three most important themes discussed when youth tried to quit smoking or the use of nicotine products, most of both the highly experienced (79%) and the less experienced (77%) municipalities answered that the most important was the social network of smokers/users of nicotine products (NP) (Figure 2).

### Danish municipalities’ experience with group-based youth-targeted cessation of smoking and nicotine products

In 78.9% of the highly experienced and 78.6% of the less experienced municipalities, nicotine product cessation was included in the smoking cessation group-based counseling (Table 3). In 88.9% of the highly experienced municipalities, counseling of youth took place separately from that of adults, compared to 63% of the less experienced municipalities. In the majority of both the highly experienced (90%) and less experienced (80.8%) municipalities, counseling took place during school hours.

Most of both the highly experienced (66.7%) and less experienced (54.5%) municipalities reported that the youth in cessation counseling stop when they are ready; there is no fixed quit date. A higher percentage of the less experienced municipalities (22.7%) reported that they only use official educational materials provided by the Danish Cancer Society, compared to the highly experienced municipalities (12.5%).

The majority in both the highly experienced (47.4%) and less experienced (59.6%) municipalities had the perception that youth were less motivated. A higher percentage of the highly experienced municipalities (68.4%) reported that youth had a higher drop-out rate from the cessation program than adults, and that the interventions had no effect on preventing drop-out (84.2%). However, 10% of the highly experienced municipalities reported that their interventions to prevent drop-out had a major effect, compared to only 1.9% of the less experienced municipality.

Danish municipalities’ experience with nicotine replacement therapy (NRT) for youth-targeted cessation of smoking cessation and nicotine products In all, 73.3% of the highly experienced and 76.0% of the less experienced municipalities recommended the use of NRT for youth (Table 4). The highly experienced municipalities reported a higher percentage of youths who use NRT, compared to the less experienced municipalities.

Also, 26.7% of the highly experienced and 31.4% of the less experienced municipalities had the perception that the required doses of NRT were higher or much higher in youths than in adults. Most of both the highly experienced (73.3%) and less experienced (72.%) had never experienced youths with overdose symptoms. A higher percentage of the highly experienced municipalities (73.7%) offered vouchers for NRT to the youths, and reported that those youths had good or very good experiences with the use of NRT (68.4%). The most frequently used types of NRT were reported to be a combination of the nicotine patch and a fast-acting NRT (Supplementary file Figure 1).

## DISCUSSION

This unique survey explored youth-targeted smoking and nicotine product cessation activities and experiences from municipality counselors across a whole nation. Our study shows that youth-targeted smoking/nicotine cessation activities are low, even in a country with very well-organized, effective, and free-of-charge cessation programs. Our study found that most municipalities in Denmark have no, very little, or little experience with smoking or nicotine product cessation assistance for adolescents and young adults aged <25 years. Almost all municipalities, even the self-perceived highly experienced ones, find it very difficult to recruit youth, and most municipalities also find it difficult to counsel them. Youth smokers/users of nicotine products are generally perceived to be less motivated to quit than adults and to have a high drop-out rate. The municipalities mostly include both cigarette and other nicotine product users in the cessation program and mostly hold youth counseling separately from adults, though a higher percentage of highly experienced municipalities reported always counseling youths in separate groups, compared to less experienced municipalities. Counseling usually takes place during school hours and NRT is often recommended. Less than half of the municipalities feel their counseling is successful in persuading youth to quit using nicotine.

### Challenges with the recruitment of youth

Previous studies have, in concordance with our study, found it difficult to recruit youths to smoking cessation interventions^19^. An article reporting on eight different youth-targeted smoking cessation programs in Scotland found that recruitment was very time-consuming and challenging, independent of the setting^20^. Our survey showed that most Danish municipalities cooperate with schools regarding recruitment. A higher percentage of the highly experienced municipalities compared to the less experienced municipalities reported sharing the responsibility for recruitment with the school. Recruitment to previous smoking cessation trials in youth has mainly taken place within educational/school settings^19^. Some previous studies recruited youth from a healthcare environment^21^, from the community^22^, or online^23^. When recruitment took place in schools, typically fewer students who smoked showed interest in enrolling^24^ than if recruitment took place in healthcare settings^25^. Recruitment to programs aimed at cessation of smokeless tobacco in youth has taken place in schools or has been community-based^26,27^. Incentives were also used in several trials^19^. The need for parental permission (when minors were included) seems to have had a negative impact on recruitment^19^.

According to a report from the national Danish Smoking Cessation Database only approximately 500 smokers aged 15–25 years (out of more than 65000 daily smokers in this age category) enrolled in the face-to-face counseling program in 2020, and the proportion of young people receiving smoking cessation support has declined in the last few years^18^. There might be several reasons for the low recruitment rate of youth. First, it might be that young people are not aware of the existence of cessation services^28^. A study from the United Kingdom found that knowledge of existing services was poor among those aged 15–19 years and that they had concerns about privacy and confidentiality^12^. Another reason could be that youths are not very interested in assistance to quit. In Denmark, only 21% of male smokers aged 16–24 years reported that they wanted support to stop smoking^13^. Further, even though the highest proportion of persons who want to quit in Denmark is found among the youngest (16–24 years), it is also known that youths are very ambivalent when it comes to quitting^29^. On the one hand, they do want to quit, but on the other, they also often deny being smokers or being addicted and they have the feeling that they can control their smoking and that it is not urgent to quit right now. This pronounced ambivalence was also mentioned in the ‘free text comments’ the counselors could add to our survey (data not shown).

### Motivating factors

The most important motivating factors for youth to quit, as perceived by the municipal counselors, were reported to be the high price of cigarettes/nicotine products, being tired of the dependence, pressure from parents, and smoking bans. This finding is in line with previous studies. Social disapproval (e.g. parents or friends disapprove smoking) has shown an association with motivation to quit and it also predicted cessation attempts^1,30^. Young persons mostly study and do not have a regular income, or only have a low income, so they spend a large proportion of their income on cigarettes/nicotine products. Several studies have found youth to be two to three times as responsive to changes in price as adults^1^. This is important knowledge if we want to motivate youth to take part in cessation activities. Most quit-smoking campaigns have long-term health consequences as the main theme and depict adults, but if we want to attract youth the themes should rather be the economic burden of smoking or the disadvantages of dependence, and they should depict young persons.

### Quit rates and drop-out

Recruitment is not the only problem. Most of the active municipalities reported that the perceived drop-out rate among youth was higher than among adults and that youth had the same or lower quit rates compared with adults. This is confirmed by data from the National Danish Smoking Cessation Database, showing that fewer young people aged <25 years complete the smoking cessation course and are self-reported smoke-free after six months^31^.

There might be various reasons for the high drop-out rates and the low quit rates. First, the above-described ambivalence and feeling that smoking cessation is not so urgent, are probably important. In our study, the counselors generally perceived youth, when they started the counseling program, to be less motivated to quit than adults. The context and meaning of smoking in adolescence are different than in adulthood. Smoking and use of nicotine products are considered more as social activities, and it might be harder to withstand social pressure. Also, adolescents’ immature brains work differently than adults’ brains when they make decisions. Youths’ actions are guided more by the emotional, risk-taking and impulsive amygdala, and less by the logical long-term consequence-thinking frontal cortex, which increases the risk of relapse^32^. Further, many young people have dual or triple use of different nicotine products which might lead to higher levels of dependence. Previous studies among youth indicate that high nicotine dependence is associated with being a dual user or user of multiple nicotine products^33^.

In our study, a higher percentage of the highly experienced municipalities reported that they do not use the official quit-smoking educational material. This result was somewhat surprising, but on reflection, the materials are targeted at adult, older smokers. Those who do not use them may have other skills; they draw on white/black boards, use videos and so on (as stated in the free-text comments by the counselors, data not shown). The most important topics discussed during the counseling, as perceived by the municipal counselors, were the social relationships of youth smokers/nicotine product users, dependence, and spare money when you quit. Long-term health consequences of use were very rarely discussed, which contrasts with the themes in the official educational quit-smoking material.

Most of the active municipalities recommend the use of NRT to youths, NRT is mostly offered free of charge, and the mean minimum age limit was 16.5 years (down to 15 years). The latter was surprising, as the use of NRT for youth below 18 years is not recommended by the Danish Health Authorities. According to the counselors, those who used it generally experienced a positive effect from it, and adverse events/overdosing were very rare. A review from 2019, based on nine randomized controlled trials in youth, found that pharmacotherapy showed an increased abstinence rate, compared with the control group, however, no efficacy was found on abstinence in the long-term^34^. A small randomized controlled trial from 2020 found that varenicline had no better effect in young smokers than placebo, but was well tolerated^35^.

### Strengths and limitations

A strength of the study is that the survey covered the whole country, and that an extremely high participation rate was achieved. One of the researchers, SBR, was the contact person and the municipalities were encouraged to contact her if they had queries. Several used this possibility, and hopefully, some misunderstandings were avoided. All the municipalities that were active in youth counseling completed the whole or most of the questionnaire depending on their level of experience. Throughout the questionnaire, the counselors could supplement with free text. The best experiences will be gathered and used to improve Danish national smoking cessation services for youth.

The study had some limitations. The questionnaire was only tested by three counselors before it was emailed to the municipalities. We do not have information on whether one, two, or several counselors completed the questionnaire together, but we know that in most municipalities there is only one counselor (in larger cities two counselors) assigned to work with youth. Answers represent the counselor’s own opinions/perceptions and could therefore have differed if another counselor from the same municipality had completed the questionnaire. Counselors’ perceptions cannot stand alone and therefore we have also performed focus group interviews with youth smokers/nicotine users attending the cessation groups.

The use of predefined answer categories when investigating motivating factors and themes entails the risk of overlooked answer categories. We tried to anticipate this by also allowing free text. We opted out of the use of the answer category ‘I don't know’ as we were afraid of extensive use when counselors were in doubt. This might in some cases have resulted in misleading answers.

Due to the limited number of respondents (a maximum of 98 municipalities) and the purpose of this study being more explorative in describing the subjective perceptions and experiences of the counselors, there was low power to detect significant predictors and to perform multivariable analyses (Supplementary file Table 1).

## CONCLUSIONS

This nationwide survey of municipal smoking cessation counselors from Denmark showed that youth-targeted smoking and nicotine product cessation activities are low, even in a country with very well-organized, effective, and free-of-charge cessation counseling programs. Less than a fifth of the municipalities had high levels of experience with youth counseling. Almost all municipalities found it very difficult to recruit youth, and most municipalities also found it difficult to counsel youth. The counselors are not trained either to counsel users of multiple novel nicotine products or to counsel youth, which might require pedagogical, rather than health-professional skills. Smoking cessation services have been designed specifically for adults and seem to have failed to meet the needs of young smokers, at least in Denmark.

There seems to be a need to develop new, youth-targeted educational quit-nicotine materials, with new topics, and which address the urgent need to include novel nicotine product and multiproduct use. We urgently need to find out how we can most effectively recruit youth to smoking/nicotine product cessation services, and what works to help youth quit both smoking and using nicotine products. Furthermore, as most young people do not want assistance to quit, we also need to stimulate unassisted cessation attempts, e.g. by increasing taxes on tobacco and nicotine products.

## Data Availability

The data supporting this research are available from the authors on reasonable request.

## References

[1] (2012). Preventing Tobacco Use Among Youth and Young Adults: A Report of the Surgeon General.

[2] Perelman J, Leão T, Kunst AE (2019). Smoking and school absenteeism among 15- to 16-year-old adolescents: a cross-section analysis on 36 European countries. Eur J Public Health.

[3] Benowitz NL (2008). Neurobiology of nicotine addiction: implications for smoking cessation treatment. Am J Med.

[4] Glenstrup S, Bast LS, Danielsen D, Andersen A, Tjørnhøj-Thomsen T (2021). Places to Smoke: Exploring Smoking-Related Practices among Danish Adolescents. Int J Environ Res Public Health.

[5] Siahpush M, Borland R, Scollo M (2003). Smoking and financial stress. Tob Control.

[6] de Looze ME, Henking C, Torsheim T, Currie DB, Weber MW, Alemán-Díaz AY (2022). The association between MPOWER tobacco control policies and adolescent smoking across 36 countries: An ecological study over time (2006-2014). Int J Drug Policy.

[7] East KA, Reid JL, Rynard VL, Hammond D (2021). Trends and Patterns of Tobacco and Nicotine Product Use Among Youth in Canada, England, and the United States From 2017 to 2019. J Adolesc Health.

[8] Raitasalo K, Bye EK, Pisinger C (2022). Single, Dual, and Triple Use of Cigarettes, e-Cigarettes, and Snus among Adolescents in the Nordic Countries. Int J Environ Res Public Health.

[9] Leslie FM (2020). Unique, long-term effects of nicotine on adolescent brain. Pharmacol Biochem Behav.

[10] Pisinger V, Thorsted A, Huber Jezek A (2019). Sundhed Og Trivsel På Gymnasiale Uddannelser 2019.

[11] Bast LS, Klitgaard MB, Kjeld SG, Jarlstrup NS, Christensen AI (2022). Use of Tobacco and Nicotine Products among Young People in Denmark-Status in Single and Dual Use. Int J Environ Res Public Health.

[12] Grimshaw G, Stanton A, Blackburn C (2003). Patterns of smoking, quit attempts and services for a cohort of 15- to 19-year-olds. Child Care Health Dev.

[13] Juel Lau C, Holm Eliasen M, Stjerne Grønkjær M (2022). Hvordan Har Du Det? Sundhedsprofil for Region Hovedstaden Og Kommuner 2021.

[14] Pedersen M, Lund L, Bast L (2022). Brug Af Røgfri Nikotinprodukt Blandt Unge.

[15] Roberts ME, Bidwell LC, Colby SM, Gwaltney CJ (2015). With others or alone? Adolescent individual differences in the context of smoking lapses. Health Psychol.

[16] Joossens L, Olefir L, Feliu A, Fernandez E (2022). The Tobacco Control Scale 2021 in Europe; WHO Collaborating Centre for Tobacco Control 2022.

[17] Neumann T, Rasmussen M, Heitmann BL, Tønnesen H (2013). Gold standard program for heavy smokers in a real-life setting. Int J Environ Res Public Health.

[18] (2021). Aktiviteter Afholdt 2020.

[19] Fanshawe TR, Halliwell W, Lindson N, Aveyard P, Livingstone-Banks J, Hartmann-Boyce J (2017). Tobacco cessation interventions for young people. Cochrane Database Syst Rev.

[20] Gnich W, Sheehy C, Amos A, Bitel M, Platt S (2008). A Scotland-wide pilot programme of smoking cessation services for young people: process and outcome evaluation. Addiction.

[21] Horn K, Dino G, Hamilton C, Noerachmanto N (2007). Efficacy of an emergency department-based motivational teenage smoking intervention. Prev Chronic Dis.

[22] Lipkus IM, McBride CM, Pollak KI, Schwartz-Bloom RD, Tilson E, Bloom PN (2004). A randomized trial comparing the effects of self-help materials and proactive telephone counseling on teen smoking cessation. Health Psychol.

[23] Patten CA, Croghan IT, Meis TM (2006). Randomized clinical trial of an Internet-based versus brief office intervention for adolescent smoking cessation. Patient Educ Couns.

[24] Mason M, Mennis J, Way T, Lanza S, Russell M, Zaharakis N (2015). Time-varying effects of a text-based smoking cessation intervention for urban adolescents. Drug Alcohol Depend.

[25] Prochaska JJ, Fromont SC, Ramo DE (2015). Gender differences in a randomized controlled trial treating tobacco use among adolescents and young adults with mental health concerns. Nicotine Tob Res.

[26] Walsh MM, Langer TJ, Kavanagh N (2010). Smokeless tobacco cessation cluster randomized trial with rural high school males: intervention interaction with baseline smoking. Nicotine Tob Res.

[27] Perry CL, Stigler MH, Arora M, Reddy KS (2009). Preventing tobacco use among young people in India: Project MYTRI. Am J Public Health.

[28] Molyneux A, Lewis S, Coleman T (2006). Designing smoking cessation services for school-age smokers: A survey and qualitative study. Nicotine Tob Res.

[29] Amos A, Wiltshire S, Haw S, McNeill A (2006). Ambivalence and uncertainty: experiences of and attitudes towards addiction and smoking cessation in the mid-to-late teens. Health Educ Res.

[30] Myers MG, MacPherson L (2008). Adolescent reasons for quitting smoking: initial psychometric evaluation. Psychol Addict Behav.

[31] Rygestopbasens Årsrapport (2020). Aktiviteter Afholdt i 2019 Med Opfølgning i 2020.

[32] Hartley CA, Somerville LH (2015). The neuroscience of adolescent decision-making. Curr Opin Behav Sci.

[33] Mohd Radzi NA, Saub R, Mohd Yusof ZY, Dahlui M, Sujak SL (2021). Nicotine Dependence among Adolescents Single and Dual Cigarette Users. Children (Basel).

[34] Myung SK, Park JY (2019). Efficacy of Pharmacotherapy for Smoking Cessation in Adolescent Smokers: A Meta-analysis of Randomized Controlled Trials. Nicotine Tob Res.

[35] Gray KM, Rubinstein ML, Prochaska JJ (2020). High-dose and low-dose varenicline for smoking cessation in adolescents: a randomised, placebo-controlled trial. Lancet Child Adolesc Health.

