# Supplementary material for: Nationwide experiences with youth-targeted smoking and nicotine product cessation
Source: Tob Prev Cessat. 2023 Aug 4;9:27. doi: 10.18332/tpc/169498 (PMC10402277; doi:10.18332/tpc/169498)
Supplement: Supplementary file 1 [file TPC-9-27-s1.pdf]

Appendix 1. The most common nicotine replacement therapy (NRT) used by youth, according to Danishmunicipality counselors that actively work with youth cessation. A national survey performed in Denmark in 2022 (N=94)

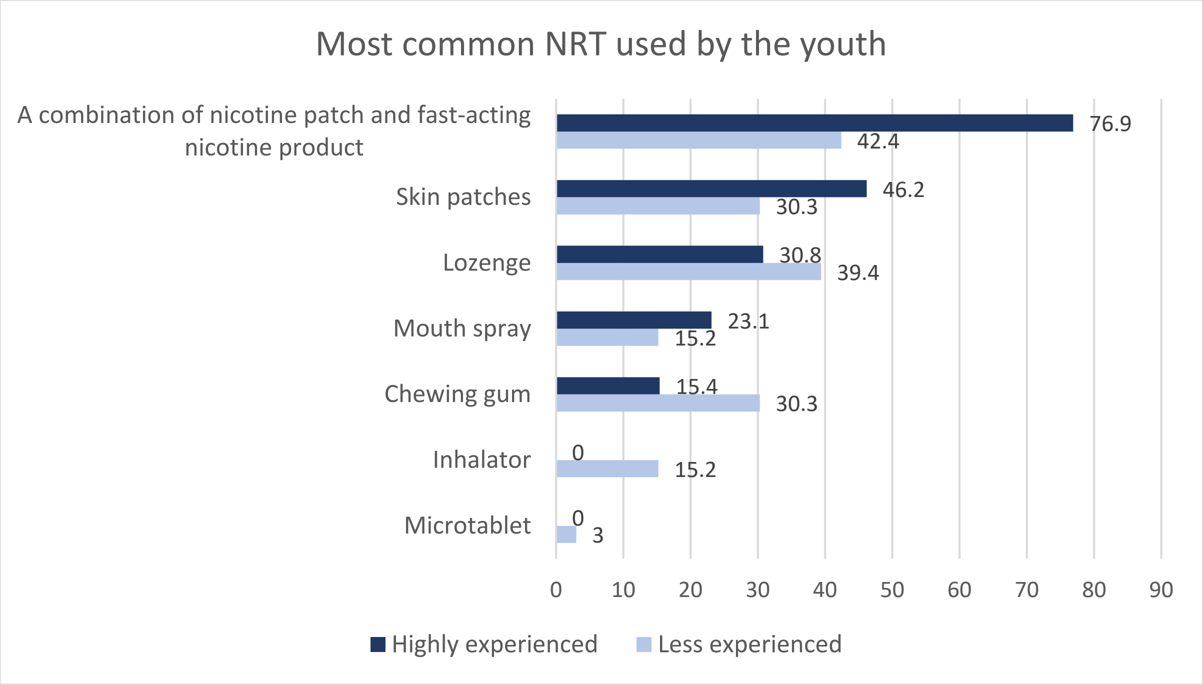

Appendix 2. Predictors for counselors' perceived success with the youth-oriented smoking and nicotine product cessation intervention among municipalities that actively work with youth cessation. A national survey performed in Denmark in 2022 (N=94)

	Number	Perceived Success with Smoking Cessation			Number	Perceived Success with Nicotine Product Cessation		
		**OR**	**95% CI**	**p**		**OR**	**95% CI**	**p**
**Municipality size**								
Small (under 70.000)	53	1			45	1		
Large (over 70.000)	18	2.06	0.70 – 6.09	0.19	17	1.49	0.48 – 4.62	0.49
**Region**								
Capital region	23	1			17	1		
South Denmark	16	1.04	0.26 – 4.13		15	1.22	0.29 – 5.13	
Zealand	9	1.14	0.22 – 5.93		8	1.83	0.33 – 10.1	
North Jutland	9	2.86	0.58 – 13.96		8	5.50	0.84 – 36.2	
Central Jutland	14	5.71	1.33 – 24.61	0.12	14	4.58	0.995 – 21.12	0.18
**Level of experience with youth-oriented cessation interventions**								
Very low/low/no	53	1			47	1		
Very high/high	18	2.06	0.70 – 6.09	0.19	15	9.58	1.94 – 47.38	**0.01**
**Counselor has attended extra courses to work with youths**								
No	27	1			22	1		
Yes, official courses at The Danish Cancer Society	39	0.54	0.42 – 43.73		35	0.38	0.13 – 1.15	
Yes, courses elsewhere	5	4.34	0.20 – 1.47	0.14	5	2.29	0.22 – 24.14	0.09
**Counseling takes part during school hours**								
No	12	1			9	1		
Yes always/ sometimes	60	1.58	0.43 – 5.81	0.49	53	1.40	0.34 – 5.80	0.64
**Smoking cessation and nicotine product cessation in the same group**								
No	13	1			13	1		
Yes	48	1.47	0.42 – 5.15	0.55	15	1.33	0.39 – 4.60	0.65
**Youths mixed with adults in the group**								
Yes	19	1			14	1		
No (youth only)	45	1.90	0.61 – 5.87	0.27	42	2.65	0.76 – 9.28	0.13
**Contact between sessions to prevent drop-out**								
No/only if they are absent from a session	15	1			12	1		
The counselor calls or sends text as a reminder before next session	51	2.86	0.80 – 10.18	0.10	47	1.89	0.52 – 6.83	0.33
**Municipality offers free NRT vouchers to youths who attends the courses**								

No	25	1			21	1		
Yes	45	2.03	0.73 – 5.66	0.17	41	1.70	0.59 – 4.93	0.33
**Counselor recommends NRT use for youths who wants to quit**								
No	17	1			14	1		
Yes	54	2.07	0.64 – 6.68	0.22	48	3.5	0.96 – 12.76	0.06
**Counselors' use educational materials #**								
Only uses the official educational materials	12	1			8	1		
Supplements with other materials	37	10.42	1.22 – 89.12		35	3.56	0.63 – 21.16	
Does not use the official educational materials	11	49.50	3.84 – 638.36	**0.01**	11	8.00	1.00 – 63.96	0.15

* NRT= nicotine replacement therapy; # provided by the Danish Cancer Society.

© Rasmussen S. K. B. et al.
